# Odontostomatological Traits in North-Eastern Italy’s Isolated Populations: An Epidemiological Cross-Sectional Study

**DOI:** 10.3390/jcm12072746

**Published:** 2023-04-06

**Authors:** Valentina Luppieri, Alessandro Pecori, Beatrice Spedicati, Riccardo Schito, Lucia Pozzan, Aurora Santin, Giorgia Girotto, Milena Cadenaro, Maria Pina Concas

**Affiliations:** 1 Institute for Maternal and Child Health—IRCCS “Burlo Garofolo”—Trieste, Via dell’Istria 65, 34137 Trieste, Italy; 2 Department of Medicine, Surgery and Health Sciences, University of Trieste, Strada di Fiume 447, 34149 Trieste, Italy

**Keywords:** epidemiologic studies, malocclusion, oral health, temporomandibular joint disorders

## Abstract

Malocclusions and temporomandibular disorders (TMDs) are oral health problems that are spread worldwide. To date, few studies focused on their prevalence and associated risk factors are available. This study aims to define the prevalence and distribution of odontostomatological traits and evaluate specific risk factors in isolated villages in north-eastern Italy, taking advantage of their environmental homogeneity. Nine hundred and forty-four participants aged six to eighty-nine years were enrolled. Thirty-one odontostomatological phenotypes, classified into five domains (airways, bad habits, extraoral and intraoral parameters, TMDs, and teeth), were evaluated. A descriptive statistical analysis was performed; mixed logistic models were used to test the relationships among the traits. According to the study’s findings, Angle’s class I was prevalent (65.3%) followed by class II malocclusion (24.3%); class III and reversed overjet were the least frequent malocclusions (10.4% and 1.8%, respectively). Temporomandibular joint (TMJ) click/noise was prevalent among TMDs (34.7%). The statistically significant (*p*-value < 0.05) risk factors were ankyloglossia for phonetic issues (OR 1.90) and bruxism for TMJ click/noise (OR 1.70) and pain (OR 2.20). Overall, this work provides a picture of the prevalence of malocclusions and TMDs in a large Italian sample and reveals risk factors to take into account in the development of preventive strategies and treatments.

## 1. Introduction

Malocclusions and temporomandibular disorders (TMDs) are estimated to be among the most prevalent oral health problems worldwide, after dental caries and periodontal disease [1,2]. Angle [3] introduced his classification of malocclusions in 1899 and in the 1980s the World Health Organization included malocclusions under the heading “handicapping dentofacial anomalies” [4]. A malocclusion is defined as “any deviation from the normal occlusion beyond the range of what is accepted as normal” [3,5]; influenced by several factors, malocclusions can be considered a manifestation of individual genetic and biological variability [6]. TMD is an umbrella term that identifies, according to the American Academy of Orofacial Pain, a group of dysfunctional and/or painful orofacial conditions involving temporomandibular joints (TMJs), masticatory muscles, and/or their associated structures [7]. TMDs are reported to be the most frequent cause of non-dental pain in the orofacial region [2,8].

Malocclusions and TMDs are multifactorial conditions [1,2,6,8,9] with a highly variable global prevalence reflecting differences among geographical areas, social and ethnic groups, and a possible lack of homogeneity in the data collection procedures of epidemiological studies [2,9,10]. The several adverse implications of malocclusions and TMDs, both at oral and systemic levels and on the affected subjects’ socio-psychological domain and quality of life, emphasize the importance of prevention, early diagnosis, and prompt treatment [10,11,12]. In this context, epidemiological studies are of great relevance in estimating the size of a problem, providing useful tools to identify the etiological factors, and setting the most correct therapy. In particular, isolated populations are an ideal sample to study multifactorial traits and diseases. In fact, isolated populations are described as geographically isolated subpopulations derived from a small number of individuals by a founding event. Isolates show higher phenotypic and genetic homogeneity in comparison with the general population [13]. When studying multifactorial traits and diseases, focusing on isolated populations represents an excellent resource to achieve homogeneity of the sample [14]. In fact, the prevalence of multifactorial traits and diseases can be variable between populations, and establishing whether this variation is due to genetics or environmental factors (specific for each population) can be challenging. Therefore, in order to accurately study the etiological factors involved, it is fundamental to collect minimally perturbed phenotypic data. In light of this, isolated populations, characterized by environmental homogeneity, can facilitate the identification of diseases’ etiological factors by reducing the variance caused by the environmental background. Several gene–environment interaction studies have been performed on isolated populations, such as the Amish [15], Icelanders [16], Northern Finns [17], and, in Italy, on Sardinians [18], Tuscans, populations of the Apulia region, Borbera Valley [19], and on communities showing evidence of isolation due to geographical, historical, linguistic, and cultural factors of a mountain area of the Friuli-Venezia Giulia (FVG) region in the Northeast of Italy [19]. Being enriched by a complex demographic history and topographic variability, Italians are thus one of the most studied European populations [19,20,21]. Regarding the study of oral health conditions in isolated populations, the literature reports some studies on dental caries and periodontal disease in Italian isolated communities [22,23,24], but a thorough search of the relevant literature highlighted that few studies on the prevalence and distribution of orthodontic traits and of other odontostomatological traits, such as breathing pattern, TMDs, and dentition, are available. Taking into account the current literature picture, the present study aims to deepen the knowledge of the epidemiology of odontostomatological traits in isolated populations of north-eastern Italy within the context of the wider project named “Friuli-Venezia Giulia (FVG) Genetic Park” [19]. In detail, the present study aims to (1) verify the prevalence and distribution of defined odontostomatological phenotypes in the whole sample and by sex and age groups and (2) analyze the relationship between the selected variables, such as the effect of bruxism on TMJ click/noise and pain. The following null hypotheses were tested: (1) the prevalence of the studied odontostomatological phenotypes would not differ according to the participants’ sex and age and (2) in the population, none of the chosen independent variables exert an effect on the dependent variable. Overall, the goal of the study is to provide a detailed epidemiological characterization of odontostomatological traits in north-eastern Italy’s isolated populations.

## 2. Materials and Methods

### 2.1. Study Population and Ethics Approval

Nine hundred forty-nine individuals from isolated villages located in the north-east of Italy (Erto–Casso, Clauzetto, Illegio, Sauris, and Val di Resia) voluntarily participated, between March 2008 and November 2008, in the dental examination organized within the “FVG Genetic Park” project, as part of a wider research program aimed at the identification of factors associated with common diseases and traits [21,23]. Inhabitants of these villages were invited to participate by public advertisements through local authorities, television, newspapers, local physicians’ involvement, and mailing. Meetings were organized to present the project. No inclusion/exclusion criteria were applied during the recruitment phase, whereas, in this study, we applied the following exclusion criteria: (1) missing data for age, sex, and village of origin (n = 1) and (2) age under six years (n = 4). The five villages considered met the criteria defining “genetic isolates” as separate geographical locations with high rates of endogamy, language barriers, few surnames, few founders, low rates of emigration and immigration, and for which genetic homogeneity was already shown [19].

All participants signed a written informed consent form to participate in the study before their enrollment; parents or legal guardians signed the informed consent form for underage participants. The ethical committee of the Institute for Maternal and Child Health—IRCCS “Burlo Garofolo” approved the study under the univocal code Prot. CE/V-78, 06/08/2007. All study procedures were performed under the ethical principles expressed by the Helsinki Declaration.

### 2.2. Data Collection

Demographic data (age, sex, and village of origin) and a detailed familial and individual medical history with more than 200 questions were collected for all the enrolled participants. Participants underwent specialist evaluations, e.g., cardiovascular, neurological, in-depth sensorial, and stomatological evaluations. Hundreds of functional parameters, including clinical biochemistry and metabolomics data, were also collected. All parameters were systematically registered by professionals according to a standardized format.

**Figure 1:**
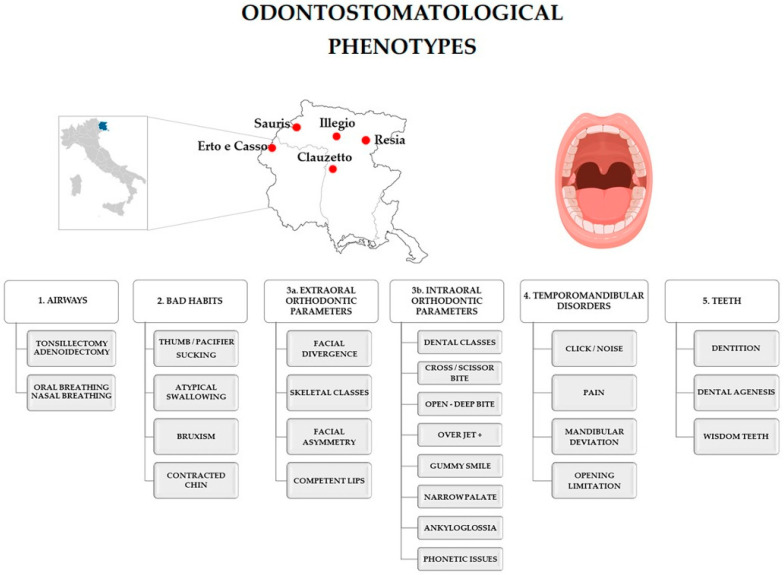
Odontostomatological phenotypes. The upper part of the figure provides a detailed map of the five isolated villages (Erto–Casso, Clauzetto, Illegio, Sauris, and Val di Resia) where the participants in the study were recruited. The lower part of the figure reports the details of the five main domains that comprise the 31 phenotypes analyzed in the study (airways, bad habits, extraoral and intraoral orthodontic parameters, temporomandibular disorder, and teeth).

With reference to the stomatological evaluation, an accurate extraoral and intraoral odontostomatological examination was performed by a team of calibrated dentists, and 31 phenotypes, reported in Figure 1 and divided into 5 main domains, were collected for each participant. The dichotomic algorithm No/Yes was followed to evaluate parameters of domain 1 (airways), 2 (bad habits), 4 (TMDs), and for specific parameters of domain 3 (a extraoral and b intraoral orthodontic parameters: mandibular deviation, competent lips, cross/scissor bite, open/deep bite, overjet, narrow palate, ankyloglossia, and phonetic issues). Regarding bad habits, thumb/pacifier sucking was evaluated by asking the participants if they used to suck their thumb/pacifier beyond the age of 18 months [25]. Atypical swallowing was clinically determined when participants contracted their perioral muscles when swallowing saliva both on command and unconsciously. Moreover, when moving the lips apart by grabbing them with a thumb and the index fingers, an anterior and/or posterior lingual interposition between the dental arches was observed during the swallowing [26]. The diagnosis of bruxism was clinically formulated, according to its main features: teeth wear and hypertrophy/pain of masticatory muscles, especially masseters, on palpation [27]. The presence of signs of wear on permanent teeth occlusal/incisal surfaces was the condictio sine qua non for the diagnosis of bruxism while the other possible clinical manifestations of bruxism such as masticatory muscles’ hypertrophy and pain on their palpation were evaluated only in participants presenting teeth wear. Bruxism was not evaluated in edentulous and in individuals with more than six missing teeth (no more than two for hemiarch). The TMJ was physically evaluated by inspection, palpation, and auscultation in the mandibular rest position and during mandibular movements [8]; TMJ pain was assessed by asking the participants if they complained of pain during mandibular movements. To evaluate mandibular deviation, participants’ face was observed in the frontal plane, and the chin position (*pogonion*) was assessed in relation to the vertical midline (*nasion-filtrum*) in the habitual occlusal position [28]. Concerning the other parameters of domain 3, facial divergence was classified as normal, anterior, or posterior, while skeletal and dental classes were classified as I, II, and III. Skeletal classes were clinically evaluated; therefore, they are referred to as “skeletal classes facial profiles”. Malocclusion diagnosis was formulated according to Angle’s criteria [3]. Angle’s classes were tested in the habitual occlusal position considering the relationship between upper and lower first permanent molars and canines; whenever one or more of these teeth were missing or had a fixed prosthetic, Angle’s classes were not assessable. Ankyloglossia was clinically determined by evaluating the lingual frenulum according to the Bristol Tongue-tie Assessment Tool (BTAT) [29].

Regarding the parameters of domain 5 (teeth), dentition was classified as primary, mixed, complete permanent (excluding wisdom teeth), partial permanent (one or more missing teeth), and edentulous. Wisdom teeth were classified as absent, present (sound, filled, or decayed), impacted, and semi-impacted. An additional X-ray examination (panoramic radiography) was performed for each participant to detect agenetic, impacted teeth and misleading carious lesions.

### 2.3. Statistical Analysis

All statistical analyses were performed using the R software version 4.1.2 (R Foundation for Statistical Computing, Vienna, Austria) and the level of significance was set to *p*-value < 0.05.

A descriptive statistical analysis including frequency and percentages was performed on categorical variables (No/Yes). Fisher’s exact test was used to evaluate the association of odontostomatological measurements between sex and age classes (five groups: 6–12; 13–17; 18–40; 41–60; and 60+ years). 

Logistic mixed effect models (lme4 package in R) were performed to evaluate associations among the selected variables. In detail, the relationships between the following pairs of variables (response and independent) were evaluated: phonetic issue and ankyloglossia, TMJ click/noise and bruxism, TMJ pain and bruxism, mandibular deviation and bruxism, and opening limitation and bruxism. All the variables were coded as the presence (value 1) or absence of the disease/issue (value 0). Age (continuous variable), sex, previous orthodontic treatment (yes, n = 140; no, n = 710), and village of origin were included as covariates in the models and, precisely, the first three were classified as fixed effects and the fourth was classified as a random effect. 

We performed post hoc power calculations for the logistic models using the WebPower package of the R software (Table S1).

## 3. Results

### 3.1. Demographic Data

The study sample (Table 1) comprised 944 participants (59.1% females) with a mean age of 45.7 ± 20.3 years. One hundred and twenty-one participants (12.8%) were underage (7.6% aged 6–12 years and 5.2% 13–17 years) while eight hundred and twenty-three (87.2%) were adults (26.2% aged 18–40 years, 33.4% 41–60 years, and 27.6% 60+ years). With regard to the village of origin, Resia was the village with the highest number of participants (n = 324) while Erto–Casso and Sauris were those with the lowest (n = 141 and n = 135, respectively).

### 3.2. Odontostomatological Phenotypes

Regarding odontostomatological phenotypes, the participants’ data on airways (domain 1), bad habits (domain 2), extraoral and intraoral orthodontic parameters (domain 3), and TMDs (domain 4) are reported in Table 2.

Regarding airways, 41.5% of the participants reported to have undergone tonsillectomy and 29.7% reported to have undergone adenoidectomy; the percentage of participants that underwent these surgeries was higher in the age group 41–60 years (53.3%) than in the other groups (*p*-value < 0.001). No sex differences were detected for both surgeries among the groups. Nasal breathing was more frequent than oral breathing (20.9% vs. 4.1%), while the remaining 75% of participants presented mixed breathing.

Concerning bad habits, pacifier sucking beyond the age of 18 months was more frequent than thumb sucking (10.9% vs. 8.7%). Differences were observed in terms of prolonged pacifier sucking frequency between the participants aged 6–12 and 60+ years (24.6% vs. 2.7%) (*p*-value < 0.001). Atypical swallowing was more frequent in the age group 6–12 years (31.5%) while the lowest prevalence was registered in the age group 18–40 years (15.4%) (*p*-value < 0.05). The overall prevalence of bruxism was 20.9% and that of a contracted chin was 13.8%.

Taking into account extraoral orthodontic traits, most participants had competent lips (91.5%) and only a small percentage showed mandibular deviation (15.4%); significant differences were found for these two parameters between participants aged 6–12 and 13–17 years (*p*-value < 0.05). Concerning facial divergence, about 50% of the participants had a normal facial divergence, while 20.0% and 30.1% had an anterior and posterior divergence, respectively. The analysis of the skeletal classes’ facial profiles revealed that class I was the most frequent (61.0%), whereas the prevalence of class II and III was similar (20.0% and 19.0%, respectively). The analysis of the frequency of the skeletal classes’ facial profiles among the age groups highlighted how the 41–60 and 60+ years groups showed the highest percentages of skeletal class I facial profile (62.1% and 64.4%, respectively), the 6–12 years group showed the highest prevalence of class II (41.8%), while class III was more prevalent in the 60+ years group (24.0%). Differences were observed for the skeletal class I facial profile between participants aged 6–12 years and older than 60 years (*p*-value < 0.001); no sex differences were found for the skeletal classes’ facial profiles among the age groups.

Within intraoral orthodontic traits, the most frequent characteristic among all age groups was overjet + (37.1%) while the less frequent characteristic was reversed overjet (1.8%). Malocclusions were evaluated both on the frontal plane, highlighting how the deep bite was more frequent than the open bite (24.5% vs. 7.5%, respectively), and on the sagittal plane, where the posterior crossbite was more frequent than the scissor bite (23.8% vs. 3.3%, respectively). Concerning molar and canine classes, class I was the most frequent (65.3% and 62.7%, respectively) followed by class II (24.3% and 27.1%, respectively) and class III (10.4% and 10.2%, respectively). Age group differences were found for molar (*p*-value < 0.05) and canine classes (*p*-value < 0.01). Molar class I was prevalent in the age group 13–17 years (75.0%), and molar classes II and III were more frequent in the age groups 6–12 years (42.5%) and 41–60 years (13.7%), respectively. Canine class I was more frequent in the age group 18–40 years (69.3%); classes II and III were more frequent in the age groups 6–12 years (58.8%) and 60+ years (15.1%), respectively. The overall prevalence of gummy smiles was 15.2% and this trait was significantly associated with sex, with females being more affected than males (18.0% vs. 10.9%, respectively) (*p*-value < 0.01). A total of 22.6% of the participants had a narrow palate and 16.9% had a short lingual frenulum; the overall prevalence of phonetic issues was 25.2%. Differences among age groups were found for both narrow palates and phonetic issues that were more frequent among underage participants in the age groups 6–12 years (48.5%) and 13–17% (39.0%) (*p*-value < 0.001), respectively.

Lastly, with regard to TMDs, 34.7% of the participants had a TMJ click/noise with a greater number of affected females than males (206 vs. 92, respectively) (*p*-value < 0.001). Differences among the age groups were detected with a higher prevalence of TMJ click/noise in the age group 41–60 years (39.3%), and the lowest prevalence was in the age group 6–12 years (11.1%) (*p*-value < 0.001). Sixty-seven participants (8.0%) complained of TMJ pain and three of them were underage; sex was significantly associated with TMJ pain with females being more affected than males (11.0% vs. 3.5%, respectively) (*p*-value < 0.001). A total of 24.6% of participants showed a mandibular deviation and differences among the age groups were found (*p*-value < 0.05). The overall prevalence of mouth-opening limitation was 3.3%.

Participants’ data on dentition, dental agenesis, and wisdom teeth are reported in Table 3.

A total of 22.3% of the participants had a complete permanent dentition (excluding wisdom teeth) while 60.0% had a partial permanent dentition with one or more missing teeth. Ninety-two individuals (10.2%) were edentulous and all of them were aged 41+ years. A higher prevalence of edentulous was found in the age group 60+ years (n = 85). The dentition type was statistically associated with age groups (*p*-value < 0.001) but not with sex. Concerning dental agenesis, 39 participants presented at least 1 agenetic tooth. The number of agenetic teeth is reported below by frequency order. The most frequently agenetic teeth were permanent upper lateral incisors (n = 27: 14 upper right and 13 upper left lateral incisors), followed by lower wisdom teeth (n = 18: ten lower left and eight lower right), upper wisdom teeth (n = 15: seven upper right and eight upper left), lower second premolars (n = 15: eight lower left and seven lower right second premolars), permanent lower central incisors (n = 7: four lower right and three lower left central incisors), and upper second premolars (n = 6: four upper right and two upper left second premolars). The less frequent agenetic teeth were instead permanent upper central incisors (n = 2), permanent lower lateral incisors (n = 2: one lower right and one lower left lateral incisors), permanent upper canines (n = 2: one upper right and one upper left canines), and permanent lower canines (n = 1: lower left canine). The corresponding deciduous tooth was still present in 8 out of the 39 participants presenting dental agenesis. Thirteen participants presented agenesis of a single tooth, fifteen presented agenesis of two teeth, and ten presented agenesis of more than two teeth (data not available for one subject). With respect to wisdom teeth, more than 70% of the participants had at least one missing wisdom tooth. Differences among the age groups were found for sound wisdom teeth that were prevalent (about 10%), in the age group 18–40 years.

### 3.3. Relationship between the Variables

In order to investigate the relationship between the selected variables, logistic mixed effect models were performed; the results of the analysis are reported in Table 4. Three significant associations were found among the five tested models. Ankyloglossia was found to be a statistically significant risk factor for phonetic issues (OR: 1.90, 95% CI: 1.21–2.98); indeed, among the participants with ankyloglossia, 27.9% (47 out of 171) had phonetic issues while 13.1% (73 out of 557) had no phonetic problems. Bruxism was detected to represent a risk factor for TMJ click/noise (OR: 1.33, 95% CI: 1.17–2.47) and pain (OR: 2.24, 95% CI: 1.22–4.11). Bruxism was not related to mandibular deviation (OR: 0.93, 95% CI: 0.61–1.41) and opening limitation (OR: 1.41, 95% CI: 0.47–3.80). In detail, 37 participants out of 184 (20.1%) with bruxism had a mandibular deviation while 112 out of 547 (20.5%) did not. Among bruxists, 26.3% (5/19) had a mouth-opening limitation while 20.3% (142/698) did not.

## 4. Discussion

The present study aimed to verify the prevalence, sex, and age distribution of de-fined odontostomatological traits as well as to test the relationship within selected odontostomatological variables, providing a detailed epidemiological characterization of odontostomatological traits in the isolated cohorts of the FVG region. Focusing on such large, isolated populations allowed us to obtain sample homogeneity and control the potential confounding etiological factors of the investigated traits.

Among all of the evaluated odontostomatological phenotypes, the most interesting findings are discussed below. Regarding malocclusions and TMDs, the data reported here are broadly in line with the literature [1,2]. Regarding dental malocclusions, in this study sample, Angle’s class I was prevalent (65.3%) followed by class II malocclusion (24.3%), while class III and reversed overjet were the least frequent malocclusions (10.4% and 1.8%), as the literature describes among Caucasians. No significant differences between males and females were found in this study, in accordance with the literature [30]. A 2018 systematic review on the global distribution of malocclusion traits, reports mean prevalence values of Angle’s classes I, II, and III and reversed overjet of 74.7%, 19.5%, 5.9%, and 4.5%, respectively [1]. The prevalence of class III malocclusion was higher, however, in comparison with the global prevalence calculated by Alhammadi and colleagues (10.3% vs. 5.9%) [1]; this difference may be explained by considering the heterogeneity of the sample evaluated by the authors which includes populations from all over the world and may also highlight a possible genetic cause in this study sample to be explored. The literature describes different patterns of skeletal classes’ distribution according to ethnicities: African and Caucasian populations show the highest prevalence of skeletal classes I and II, respectively, whereas Asians show the highest prevalence of class III, suggesting a genetic contribution to the malocclusions’ etiology [1]. To date, several genes (e.g., *COL1A1*, *FGFR2*, *MATN1,* and *MYO1H*) and pathways (e.g., insulin receptor and *FGFR2* signaling cascades) that are involved in craniofacial development have been identified [31]. However, due to the multifactorial etiology of malocclusions, more studies aimed at deepening the knowledge of the molecular and genetic mechanisms underlying their etiology are needed, with the final aim of better understanding these traits and developing personalized treatment strategies. As for the other malocclusion traits, this study’s findings are in line with the literature [1,30]. Increased overjet was more frequent than reversed overjet (37.1% vs. 1.8%, respectively); deep bite prevalence was higher that open bite (24.5% vs. 7.5%, respectively), as well as posterior cross bite in comparison with scissor bite (23.8% vs. 3.3%, respectively).

Regarding TMDs, the prevalence of TMJ click/noise and pain, clinical manifestations of disc displacement with reduction (DDwR), was higher in the adults than in the underage participants (37.1% vs. 17.2%, respectively) and in females rather than in males, as reported in a 2021 systematic review and meta-analysis [2]. According to Valesan and colleagues, the overall mean prevalence of TMDs is approximately 31% for adults/the elderly and 11% for children/adolescents, and DDwR is the most frequent TMD with a higher global mean prevalence in adults than in children (25.9% vs. 7.4%, respectively) [2].

Concerning permanent dentition, the fact that most participants had a partial permanent dentition (60.0%) with one or more missing teeth, excluding wisdom teeth, and only 22.3% had a complete permanent dentition, may reflect the possible reduced attention of the participants to proper at-home oral hygiene as well as non-conservative dental treatments. The majority of the participants that had a complete permanent dentition were females (133 vs. 69). The prevalence of dental agenesis in the study sample was 6.2%; permanent upper lateral incisors followed by second premolars were the most frequently missing teeth as reported in the literature, which estimates a global prevalence of hypodontia ranging from 1.6 to 6.9% depending on the studied population [32]. Agenesis of third molars is considered a physiologic finding or an evolutionary adaptation of the dentition rather than a developmental disturbance [33], unlike other teeth agenesis.

Logistic mixed models’ findings on the relationship between variables highlighted that bruxism resulted as a risk factor for TMJ click/noise and pain as the literature suggests. To date, however, the exact relationship among these disorders is currently controversial due to the complexity of their etiology and diagnosis [27,34]. Ankyloglossia was found to be a risk factor for phonetic issues, while, according to the literature, there is no clear connection between a short lingual frenulum and speech problems, mainly due to a lack of a uniform lingual frenulum grading system and standardized clinical studies evaluating the effects of surgical treatment on speech articulation [35].

This study presents some limitations that need to be considered. Firstly, this is a cross-sectional study; therefore, to develop a better model to deepen the knowledge of specific phenotypes, such as malocclusions and TMDs, a prospective cohort study and an interventional study would be needed. In addition, when discussing this study’s findings, a detailed comparison with the literature data of the prevalence and distribution of all the studied phenotypes was not possible, due to wide diversity in the samples’ composition as well as in the outcome measurements of already published epidemiological studies. Particularly, in the literature, epidemiological studies on the distribution of odontostomatological traits in both pediatric and adult populations are heterogeneous due to differences in samples’ composition (different ethnicities, ages, and number of participants) and outcome measurements [1,2,6,8,9,10]. For these reasons, standardized epidemiological studies on odontostomatological traits are needed in order to obtain a reliable picture of their prevalence and distribution among different ethnicities and ages. Knowing the type and distribution of some orthodontic characteristics such as malocclusions and TMDs stratified by age may be useful for educational purposes as well as in determining and directing health policies regarding the treatment of odontostomatological needs and the implementation of specialists’ skills. Nowadays, orthodontic treatment is receiving great attention from both specialists and patients due to the psychological and social impact of malocclusions. According to a 2020 systematic review and meta-analysis [30], more than half of children and adolescents worldwide present a malocclusion. Since malocclusions do not self-correct with age but instead tend to get worse, knowing their prevalence and distribution in children may help in performing specific therapy plans to treat them early, thus improving their quality of life and reducing the care burden in adults; early pediatric dental visits and preventive interventions are therefore the key to preserving oral health. The future prospects of this study include performing an over-ten-years-later follow-up and deepening the knowledge on selected odontostomatological phenotypes (i.e., ankyloglossia, dental agenesis, and skeletal classes) through a combined approach of family-based studies and genetic analyses.

## 5. Conclusions

Overall, this work provides a picture of the distribution of malocclusions and TMDs in a large sample of almost one thousand subjects. This study was not focused on a specific target of individuals (i.e., children or patients from dental clinics), but rather people of all ages were included, from children to adolescents, adults, and the elderly. Moreover, including such a large general population allowed us to collect data about individuals rarely described in the literature. Indeed, most published studies are about children and adolescents or involve adult males who have served in the military [1]. Only a few studies concern adult females and people older than 40 [1]. This approach allowed the description of detailed distributions across age classes and sex, which is helpful for further comparison studies in general populations or specific groups of individuals. The findings described here reveal the associations between variables, such as ankyloglossia and phonetic issues, bruxism and click/noise, bruxism, and pain. As already discussed, the exact relationships among these disorders and the possible risk factors are currently controversial. However, this study provides further confirmation of these relationships in a large sample, highlighting the importance to take into account these risk factors in preventive strategies and treatments.

## Figures and Tables

**Table 1:** Demographic characteristics of the participants. The total number of participants, the number of males and females, and the number of participants from each of the five isolated villages are reported. Data are presented as numbers and percentages (in brackets), both for the total sample (n = 944), and divided into five age groups. M: males; F: females; Yrs: years.

	Total	6–12 yrs	13–17 yrs	18–40 yrs	41–60 yrs	60+ yrs
	944	72	49	247	315	261
Gender						
M	386 (40.9)	28 (38.9)	20 (40.8)	91 (36.8)	129 (41)	118 (45.2)
F	558 (59.1)	44 (61.1)	29(59.2)	156 (63.2)	186 (59)	143 (54.8)
Village of origin						
Erto–Casso	141 (14.9)	15 (20.8)	13 (26.5)	33 (13.4)	46 (14.6)	34 (13.0)
Clauzetto	169 (17.9)	10 (13.9)	2 (4.1)	34 (13.8)	65 (20.6)	58 (22.2)
Illegio	175 (18.5)	14 (19.4)	12 (24.5)	51 (20.6)	55 (17.5)	43 (16.5)
Resia	324 (34.3)	24 (33.3)	17 (34.7)	92 (37.2)	107 (34.0)	84 (32.2)
Sauris	135 (14.4)	9 (12.5)	5 (10.2)	37 (15.0)	42 (13.3)	42 (16.1)

**Table 2:** Characteristics of the participants’ odontostomatological measures of domains 1 (airways), 2 (bad habits), 3 (extraoral and intraoral orthodontic parameters), and 4 (Temporomandibular disorders). The values indicate the presence of the parameters in numbers and percentage (in brackets) for all samples (All), females (F), males (M), and for age groups. Yrs: years. The *p*-value columns refer to the difference in sex and age classes obtained by Fisher’s exact test.

			All	Sex			Age Classes					
Domain	Parameter			F	M	*p*-Value	6–12 yrs	13–17 yrs	18–40 yrs	41–60 yrs	60+ yrs	*p*-Value
**1. Airways**	**Tonsillectomy (n ^a^ 895)**		371 (41.5)	222 (41.8)	149 (40.9)		26 (37.7)	12 (27.9)	65 (28.1)	161 (53.3)	107 (42.8)	***
	**Adenoidectomy (n 893)**		265 (29.7)	153 (28.9)	112 (30.8)		25 (36.2)	13 (30.2)	60 (26.0)	103 (34.3)	64 (25.6)	
	**Nasal breathing (n 829)**		173 (20.9)	100 (20.4)	73 (21.5)		16 (28.6)	9 (22.5)	46 (21.3)	59 (20.9)	43 (18.3)	
	**Oral breathing (n 834)**		34 (4.1)	22 (4.4)	12 (3.5)		1 (1.7)	0 (0.0)	5 (2.3)	15 (5.3)	13 (5.5)	
**2. Bad habits**	**Pacifier sucking (n 829)**		90 (10.9)	61 (12.3)	29 (8.7)		16 (24.6)	10 (24.4)	38 (17.4)	20 (7.1)	6 (2.7)	***
	**Thumb sucking (n 865)**		75 (8.7)	42 (8.1)	33 (9.5)		9 (13.4)	7 (16.7)	22 (9.7)	23 (7.7)	14 (6.1)	
	**Atypical swallowing** **(n 657)**		125 (19.0)	74 (19.0)	51 (19.0)		17 (31.5)	7 (18.9)	32 (15.4)	40 (16.8)	29 (24.2)	*
	**Bruxism (n 772)**		161 (20.9)	98 (21.3)	63 (20.2)		9 (14.1)	5 (11.9)	43 (18.8)	67 (23.4)	37 (24.5)	
	**Contracted chin (n 814)**		112 (13.8)	71 (14.7)	41 (12.3)		12 (20.3)	6 (16.2)	27 (12.3)	46 (16.6)	21 (9.5)	
**3a. Extraoral orthodontic parameters**	**Competent lips (n 792)**		725 (91.5)	434 (92.1)	291 (90.7)		56 (83.6)	43 (95.6)	229 (94.2)	268 (93.4)	129 (86.0)	**
	**Mandibular deviation** **(n 852)**		131 (15.4)	77 (15.3)	54 (15.5)		4 (6.2)	8 (20.5)	33 (14.4)	58 (20.1)	28 (12.1)	*
	**Facial divergence** **(n 874)**											
		Normal	436 (49.9)	250 (48.4)	186 (52.1)		33 (50.8)	23 (48.9)	117 (49.8)	154 (51.5)	109 (47.8)	
		Anterior	175 (20.0)	100 (19.3)	75 (21.0)		12 (18.4)	17 (36.2)	45 (19.1)	61 (20.4)	40 (17.5)	
		Posterior	263 (30.1)	167 (32.3)	96 (26.9)		20 (30.8)	7 (14.9)	73 (31.1)	84 (28.1)	79 (34.7)	
	**Skeletal classes facial profiles (n 574)**											***
		I	350 (61.0)	214 (61.3)	136 (60.4)		22 (51.2)	18 (56.2)	96 (60.4)	131 (62.1)	83 (64.4)	
		II	115 (20.0)	72 (20.6)	43 (19.1)		18 (41.8)	8 (25.0)	37 (23.3)	37 (17.5)	15 (11.6)	
		III	109 (19.0)	63 (18.1)	46 (20.5)		3 (7.0)	6 (18.8)	26 (16.3)	43 (20.4)	31 (24.0)	
**3b. Intraoral orthodontic parameters**	**Overjet +** **(n 663)**		246 (37.1)	163 (40.9)	83 (31.4)		27 (46.6)	13 (31.7)	78 (35.0)	100 (40.8)	28 (29.2)	
	**Reversed overjet (n 663)**		12 (1.8)	6 (1.5)	6 (2.3)		0 (0.0)	0 (0.0)	4 (1.8)	6 (2.5)	2 (2.1)	
	**Posterior cross bite (n 694)**		165 (23.8)	96 (23.2)	69 (24.6)		11 (18.3)	10 (25.6)	62 (27.9)	69 (25.8)	13 (12.3)	*
	**Scissor bite** **(n 699)**		22 (3.3)	12 (3.0)	10 (3.7)		4 (6.7)	2 (5.3)	8 (3.7)	5 (2.0)	3 (2.9)	
	**Open bite** **(n 677)**		51 (7.5)	32 (7.9)	19 (7.0)		4 (6.5)	3 (7.5)	21 (9.5)	19 (7.4)	4 (4.2)	
	**Deep bite** **(n 681)**		167 (24.5)	99 (24.3)	68 (24.9)		21 (33.3)	9 (23.1)	56 (24.9)	67 (26.2)	14 (14.3)	*
	**Molar class (n 288)**											*
		I	188 (65.3)	118 (65.9)	70 (64.2)		25 (53.2)	30 (75.0)	96 (70.6)	28 (54.9)	9 (64.3)	
		II	70 (24.3)	47 (26.3)	23 (21.1)		20 (42.5)	6 (15.0)	24 (17.6)	16 (31.4)	4 (28.6)	
		III	30 (10.4)	14 (7.8)	16 (14.7)		2 (4.3)	4 (10.0)	16 (11.8)	7 (13.7)	1 (7.1)	
	**Canine class (n 450)**											**
		I	282 (62.7)	156 (60.2)	126 (66.0)		7 (41.2)	22 (64.7)	124 (69.3)	93 (55.7)	36 (67.9)	
		II	122 (27.1)	78 (30.1)	44 (23.0)		10 (58.8)	9 (26.5)	36 (20.1)	58 (34.7)	9 (17.0)	
		III	46 (10.2)	25 (9.7)	21 (11.0)		0 (0.0)	3 (8.8)	19 (10.6)	16 (9.6)	8 (15.1)	
	**Gummy smile (n 759)**		115 (15.2)	82 (18.0)	33 (10.9)	**	9 (13.4)	7 (16.3)	44 (19.3)	42 (15.1)	13 (9.1)	
	**Narrow palate (n 875)**		198 (22.6)	121 (23.4)	77 (21.6)		33 (48.5)	13 (30.2)	55 (23.8)	75 (25.3)	22 (9.3)	***
	**Ankyloglossia (n 877)**		148 (16.9)	88 (16.9)	60 (16.9)		12 (18.5)	7 (16.3)	35 (15.2)	49 (16.3)	45 (19.0)	
	**Phonetic issues (n 753)**		190 (25.2)	104 (23.3)	86 (28.0)		16 (26.7)	16 (39.0)	38 (17.0)	62 (22.6)	58 (37.4)	**
**4. TMDs ^b^**	**Click/noise** **(n 859)**		298 (34.7)	206 (40.2)	92 (26.5)	***	7 (11.1)	10 (23.3)	77 (33.9)	114 (39.3)	90 (38.1)	***
	**Pain (n 842)**		67 (8.0)	55 (11.0)	12 (3.5)	***	0 (0.0)	3 (7.0)	14 (6.3)	29 (10.2)	21 (9.0)	*
	**Mandibular deviation** **(n 847)**		208 (24.6)	136 (27.0)	72 (20.9)		7 (11.1)	7 (17.1)	57 (25.6)	85 (29.6)	52 (22.3)	*
	**Opening limitation** **(n 829)**		27 (3.3)	17 (3.5)	10 (2.9)		1 (1.6)	1 (2.4)	8 (3.6)	7 (2.5)	10 (4.4)	*

^a^ Number of individuals for which data are available. ^b^ TMDs = temporomandibular disorders. * *p*-value <0.05; ** *p*-value <0.01; *** *p*-value <0.001; in blank space: *p*-value not significant.

**Table 3:** Characteristics of the participants’ odontostomatological measures of domain 5 (teeth). The values indicate the presence of the parameters in numbers and percentage (in brackets) for all samples (All), females (F), males (M)m and for age groups. Yrs: years. The *p*-value columns refer to the difference in sex and age classes obtained by Fisher’s exact test.

			All	Sex			Age Classes					
Domain	Parameter			F	M	*p*-Value	6–12 yrs	13–17 yrs	18–40 yrs	41–60 yrs	60+ yrs	*p*-Value
	**Dentition** **(n ^a^ 904)**											***
**5. Teeth**		**Primary**	2 (0.2)	1 (0.2)	1 (0.3)		2 (2.9)	0 (0.0)	0 (0.0)	0 (0.0)	0 (0.0)	
		**Mixed**	66 (7.3)	39 (7.3)	27 (7.3)		62 (91.2)	4 (9.1)	0 (0.0)	0 (0.0)	0 (0.0)	
		**Complete permanent**	202 (22.3)	133 (24.8)	69 (18.8)		4 (5.9)	31 (70.5)	133 (55.0)	31 (10.3)	3 (1.2)	
		**Partial permanent**	542 (60.0)	306 (57.1)	236 (64.1)		0 (0.0)	9 (20.5)	109 (45.0)	263 (87.4)	161 (64.7)	
		**Edentulous**	92 (10.2)	57 (10.6)	35 (9.5)		0 (0.0)	0 (0.0)	0 (0.0)	7 (2.3)	85 (34.1)	
	**Dental agenesis (n 629)**	**All teeth**	39 (6.2)	28 (7.4)	11 (4.4)		6 (10.2)	6 (17.1)	20 (9.9)	7 (3.0)	0 (0.0)	***
		**Upper lateral incisors**	16 (2.5)	11 (2.9)	5 (2.0)		3 (5.1)	2 (5.7)	9 (4.4)	2 (0.9)	0 (0.0)	*
		**Second premolars**	15 (2.4)	11 (3.2)	4 (1.2)		3 (5.1)	2 (5.7)	9 (4.4)	1 (0.4)	0 (0.0)	**
		Wisdom teeth										
	**Upper right** **(n 801)**					**						***
		**Sound**	55 (6.9)	21 (4.3)	34 (10.7)		0 (0.0)	0 (0.0)	24 (13.0)	22 (8.5)	9 (3.7)	
		**Decayed**	18 (2.2)	7 (1.4)	11 (3.5)		0 (0.0)	0 (0.0)	15 (8.2)	3 (1.2)	0 (0.0)	
		**Filled**	41 (5.1)	26 (5.4)	15 (4.7)		0 (0.0)	0 (0.0)	13 (7.1)	22 (8.5)	6 (2.5)	
		**Missing**	596 (74.4)	373 (77.1)	223 (70.3)		65 (91.5)	12 (27.3)	96 (52.2)	201 (77.6)	222 (91.4)	
		**Impacted**	88 (11.0)	55 (11.4)	33 (10.4)		6 (8.5)	31 (70.5)	35 (19.0)	10 (3.9)	6 (2.5)	
		**Semi-impacted**	3 (0.4)	2 (0.4)	1 (0.3)		0 (0.0)	1 (2.3)	1 (0.5)	1 (0.4)	0 (0.0)	
	**Upper left** **(n 802)**											***
		**Sound**	54 (6.7)	23 (4.8)	31 (9.7)		0 (0.0)	0 (0.0)	32 (16.8)	15 (5.8)	7 (3.0)	
		**Decayed**	11 (1.4)	7 (1.4)	4 (1.3)		0 (0.0)	0 (0.0)	5 (2.6)	4 (1.5)	2 (0.8)	
		**Filled**	19 (2.4)	9 (1.9)	10 (3.1)		0 (0.0)	0 (0.0)	7 (3.7)	8 (3.1)	4 (1.7)	
		**Missing**	633 (78.9)	396 (82.0)	237 (74.3)		65 (91.5)	13 (29.5)	118 (61.8)	219 (84.6)	218 (92)	
		**Impacted**	82 (10.2)	46 (9.5)	36 (11.3)		6 (8.5)	30 (68.2)	28 (14.7)	12 (4.6)	6 (2.5)	
		**Semi-impacted**	3 (0.4)	2 (0.4)	1 (0.3)		0 (0.0)	1 (2.3)	1 (0.5)	1 (0.4)	0 (0.0)	
	**Lower right** **(n 803)**					*						***
		**Sound**	67 (8.3)	28 (5.7)	39 (12.4)		0 (0.0)	0 (0.0)	32 (15.7)	24 (9.6)	11 (4.7)	
		**Decayed**	11 (1.4)	7 (1.4)	4 (1.3)		0 (0.0)	0 (0.0)	7 (3.4)	3 (1.2)	1 (0.4)	
		**Filled**	46 (5.7)	26 (5.3)	20 (6.3)		0 (0.0)	0 (0.0)	10 (4.9)	30 (12.0)	6 (2.6)	
		**Missing**	584 (72.7)	372 (76.2)	212 (67.3)		65 (91.5)	11 (25.0)	111 (54.4)	185 (73.7)	212 (91.0)	
		**Impacted**	85 (10.6)	51 (10.5)	34 (10.8)		6 (8.5)	31 (70.5)	36 (17.6)	9 (3.6)	3 (1.3)	
		**Semi-impacted**	10 (1.2)	4 (0.8)	6 (1.9)		0 (0.0)	2 (4.5)	8 (3.9)	0 (0.0)	0 (0.0)	
	**Lower left** **(n 815)**					**						***
		**Sound**	61 (7.5)	27 (5.5)	34 (10.5)		0 (0.0)	0 (0.0)	25 (12.4)	28 (10.6)	8 (3.4)	
		**Decayed**	16 (2.0)	6 (1.2)	10 (3.1)		0 (0.0)	0 (0.0)	8 (4.0)	7 (2.7)	1 (0.4)	
		**Filled**	51 (6.3)	39 (7.9)	12 (3.7)		0 (0.0)	0 (0.0)	19 (9.4)	26 (9.8)	6 (2.6)	
		**Missing**	589 (72.3)	364 (74.1)	225 (69.4)		65 (91.5)	12 (27.3)	100 (49.5)	197 (74.6)	215 (91.9)	
		**Impacted**	84 (10.3)	48 (9.8)	36 (11.1)		6 (8.5)	30 (68.2)	39 (19.3)	5 (1.9)	4 (1.7)	
		**Semi-impacted**	14 (1.7)	7 (1.4)	7 (2.2)		0 (0.0)	2 (4.5)	11 (5.4)	1 (0.4)	0 (0.0)	

^a^ Number of individuals for which data are available. * *p*-value <0.05; ** *p*-value <0.01; *** *p*-value <0.001; in blank space: *p*-value not significant.

**Table 4:** Results of the logistic mixed models. The data are the odds ratio (OR) and 95% confidence interval (95% CI). Statistically significant betas are indicated in bold. All of the models were adjusted for age, gender, and previous orthodontic treatment (OT) (as fixed effect) and village of origin (as random effect).

Response Variable	Risk Factor	OR (95% CI)	Gender—OR (95% CI)	Age—OR (95% CI)	OT—OR (95% CI)
Phonetic Issues	Ankyloglossia	**1.90 (1.21–2.98)**	1.31 (0.90–1.90)	1.01 (1.00–1.02)	0.88 (0.51–1.15)
Click/noise	Bruxism	**1.70 (1.17–2.47)**	0.56 (0.40–0.78)	1.02 (1.01–1.03)	0.93 (0.60–1.45)
Pain	Bruxism	**2.20 (1.20–4.02)**	0.26 (0.12–0.55)	1.02 (1.01–1.04)	1.19 (0.55–2.59)
Mandibular deviation	Bruxism	0.93 (0.61–1.41)	0.62 (0.44–0.89)	1.01 (1.00–1.02)	0.91 (0.56–1.48)
Opening limitation	Bruxism	1.33 (0.47–3.80)	0.90 (0.35–2.36)	1.00 (0.98–1.03)	1.74 (0.58–5.27)

## Data Availability

Data are available upon request to the corresponding author.

## Supplementary Materials

The following supporting information can be downloaded at: https://www.mdpi.com/article/10.3390/jcm12072746/s1. Table S1: Post-hoc power calculation for the logistic models.

## References

[1] Alhammadi M.S., Halboub E., Fayed M.S., Labib A., El-Saaidi C. (2018). Global Distribution of Malocclusion Traits: A Systematic Review. Dent. Press J. Orthod..

[2] Valesan L.F., Da-Cas C.D., Réus J.C., Denardin A.C.S., Garanhani R.R., Bonotto D., Januzzi E., de Souza B.D.M. (2021). Prevalence of Temporomandibular Joint Disorders: A Systematic Review and Meta-Analysis. Clin. Oral Investig..

[3] Angle E.H. (1899). Classification of Malocclusion. Dent. Cosm. Mon. Rec. Dent. Sci..

[4] World Health Organization (2013). Oral Health Surveys: Basic Methods.

[5] Balachandran P., Janakiram C. (2021). Prevalence of Malocclusion among 8-15 Years Old Children, India—A Systematic Review and Meta-Analysis. J. Oral Biol. Craniofac. Res..

[6] Todor B.I., Scrobota I., Todor L., Lucan A.I., Vaida L.L. (2019). Environmental Factors Associated with Malocclusion in Children Population from Mining Areas, Western Romania. Int. J. Environ. Res. Public Health.

[7] (2015). Orofacial Pain: Guidelines for Assessment, Diagnosis, and Management. Stomatol. EDU J..

[8] Kapos F.P., Exposto F.G., Oyarzo J.F., Durham J. (2020). Temporomandibular Disorders: A Review of Current Concepts in Aetiology, Diagnosis and Management. Oral Surg..

[9] Devanna R., Felemban N.H., Althomali Y., Battepati P.M., Ali Alfawzan A., Gupta P. (2021). Prevalence of Malocclusion among Children of the Kingdom of Saudi Arabia—A Systematic Review and Meta-Analysis. Saudi Dent. J..

[10] Caccianiga P., Mantovani L.G., Baldoni M., Caccianiga G. (2022). Distribution of Malocclusion Traits in the Pediatric Population of Milan: An Observational Study. Int. J. Environ. Res. Public Health.

[11] Kragt L., Dhamo B., Wolvius E.B., Ongkosuwito E.M. (2016). The Impact of Malocclusions on Oral Health-Related Quality of Life in Children—A Systematic Review and Meta-Analysis. Clin. Oral Investig..

[12] Pigozzi L.B., Pereira D.D., Pattussi M.P., Moret-Tatay C., Irigaray T.Q., Weber J.B.B., Grossi P.K., Grossi M.L. (2021). Quality of Life in Young and Middle Age Adult Temporomandibular Disorders Patients and Asymptomatic Subjects: A Systematic Review and Meta-Analysis. Health Qual. Life Outcomes.

[13] Hatzikotoulas K., Gilly A., Zeggini E. (2014). Using population isolates in genetic association studies. Brief Funct. Genom..

[14] Kristiansson K., Naukkarinen J., Peltonen L. (2008). Isolated Populations and Complex Disease Gene Identification. Genome Biol..

[15] Puffenberger E.G. (2003). Genetic Heritage of the Old Order Mennonites of Southeastern Pennsylvania. Am. J. Med. Genet. C Semin. Med. Genet..

[16] Price A.L., Helgason A., Palsson S., Stefansson H., St Clair D., Andreassen O.A., Reich D., Kong A., Stefansson K. (2009). The Impact of Divergence Time on the Nature of Population Structure: An Example from Iceland. PLoS Genet..

[17] Peltonen L., Jalanko A., Varilo T. (1999). Molecular Genetics of the Finnish Disease Heritage. Hum. Mol. Genet..

[18] Orrù S., Thomas G., Loizedda A., Cox D.W., Contu L. (1997). 24 Bp Deletion and Ala1278 to Val Mutation of the ATP7B Gene in a Sardinian Family with Wilson Disease. Hum. Mutat..

[19] Esko T., Mezzavilla M., Nelis M., Borel C., Debniak T., Jakkula E., Julia A., Karachanak S., Khrunin A., Kisfali P. (2013). Genetic Characterization of Northeastern Italian Population Isolates in the Context of Broader European Genetic Diversity. Eur. J. Hum. Genet. EJHG.

[20] Xue Y., Mezzavilla M., Haber M., McCarthy S., Chen Y., Narasimhan V., Gilly A., Ayub Q., Colonna V., Southam L. (2017). Enrichment of Low-Frequency Functional Variants Revealed by Whole-Genome Sequencing of Multiple Isolated European Populations. Nat. Commun..

[21] Cocca M., Barbieri C., Concas M.P., Robino A., Brumat M., Gandin I., Trudu M., Sala C.F., Vuckovic D., Girotto G. (2020). A Bird’s-Eye View of Italian Genomic Variation through Whole-Genome Sequencing. Eur. J. Hum. Genet. EJHG.

[22] Navarra C.O., Robino A., Pirastu N., Bevilacqua L., Gasparini P., Di Lenarda R., Crovella S. (2016). Caries and Innate Immunity: DEFB1 Gene Polymorphisms and Caries Susceptibility in Genetic Isolates from North-Eastern Italy. Caries Res..

[23] Zupin L., Moura Rodrigues R., Navarra C.O., Bevilacqua L., Catamo E., Di Lenarda R., Gasparini P., Crovella S., Robino A. (2019). Association of LTA Gene Haploblock with Periodontal Disease in Italian Adults. J. Periodontal Res..

[24] Mezzavilla M., Navarra C.O., Di Lenarda R., Gasparini P., Bevilacqua L., Robino A. (2021). Runs of Homozygosity Are Associated with Staging of Periodontitis in Isolated Populations. Hum. Mol. Genet..

[25] American Academy of Pediatric Dentistry (2022). Policy on pacifiers. The Reference Manual of Pediatric Dentistry.

[26] Melsen B., Attina L., Santuari M., Attina A. (1987). Relationships between swallowing pattern, mode of respiration, and development of malocclusion. Angle Orthod..

[27] Melo G., Duarte J., Pauletto P., Porporatti A.L., Stuginski-Barbosa J., Winocur E., Flores-Mir C., De Luca Canto G. (2019). Bruxism: An Umbrella Review of Systematic Reviews. J. Oral Rehabil..

[28] Proffit W.R. (2001). Ortodonzia Moderna.

[29] Ingram J., Johnson D., Copeland M., Churchill C., Taylor H., Emond A. (2015). The development of a tongue assessment tool to assist with tongue-tie identification. Arch. Dis. Child. Fetal Neonatal Ed..

[30] Lombardo G., Vena F., Negri P., Pagano S., Barilotti C., Paglia L., Colombo S., Orso M., Cianetti S. (2020). Worldwide prevalence of malocclusion in the different stages of dentition: A systematic review and meta-analysis. Eur. J. Paediatr. Dent..

[31] Gershater E., Li C., Ha P., Chung C.H., Tanna N., Zou M., Zheng Z. (2021). Genes and Pathways Associated with Skeletal Sagittal Malocclusions: A Systematic Review. Int. J. Mol. Sci..

[32] Al-Ani A.H., Antoun J.S., Thomson W.M., Merriman T.R., Farella M. (2017). Hypodontia: An Update on Its Etiology, Classification, and Clinical Management. BioMed Res. Int..

[33] Scheiwiller M., Oeschger E.S., Gkantidis N. (2020). Third Molar Agenesis in Modern Humans with and without Agenesis of Other Teeth. PeerJ.

[34] Jiménez-Silva A., Peña-Durán C., Tobar-Reyes J., Frugone-Zambra R. (2017). Sleep and Awake Bruxism in Adults and Its Relationship with Temporomandibular Disorders: A Systematic Review from 2003 to 2014. Acta Odontol. Scand..

[35] Wang J., Yang X., Hao S., Wang Y. (2022). The Effect of Ankyloglossia and Tongue-Tie Division on Speech Articulation: A Systematic Review. Int. J. Paediatr. Dent..

