# Supplementary material for: Odontostomatological Traits in North-Eastern Italy’s Isolated Populations: An Epidemiological Cross-Sectional Study
Source: J Clin Med. 2023 Apr 6;12(7):2746. doi: 10.3390/jcm12072746 (PMC10095173; doi:10.3390/jcm12072746)
Supplement: Supplementary file 1 [file jcm-12-02746-s001.zip › jcm-2274586-supplementary.pdf]

Supplementary Table S1. Post-hoc power calculation for the logistic models.

Response variable (Y)	Risk factor (X)	Sample size	P(Y=1|X=0)[a]	P(Y=1|X=1)[b]	Significance level	Power[c]
Phonetic issues	Ankyloglossia	728	0.204	0.392	0.05	**1**
Click/Noise	Bruxism	737	0.321	0.457	0.05	**0.96**
Pain	Bruxism	725	0.059	0.128	0.05	**0.88**
Mandibular deviation	Bruxism	731	0.253	0.248	0.05	**0.05**
Opening limitation	Bruxism	717	0.025	0.034	0.05	**0.11**

[a] The probability of having positive outcome (Y=1) in the absence of the risk factor (X=0).

[b] The probability of having positive outcome (Y=1) in the presence of the risk factor (X=1).

[c] Calculated using the WebPower package of the R software.
